# Development and validation of SIRT3-related nomogram predictive of overall survival in patients with serous ovarian cancer

**DOI:** 10.1186/s13048-019-0524-2

**Published:** 2019-05-21

**Authors:** Jun Li, Huiran Yue, Hailin Yu, Xin Lu, Xiaohong Xue

**Affiliations:** 1 Department of Gynecology, Obstetrics and Gynecology Hospital, Fudan University, No.419, Fangxie Road, Shanghai, 200011 China; 2 Shanghai Key Laboratory of Female Reproductive Endocrine Related Diseases, Shanghai, 200011 China

**Keywords:** Ovarian cancer, Sirtuin, SIRT3, Nomogram, Prognosis

## Abstract

**Objective:**

Our aim is to analyzed the expression pattern of sirtuin(SIRT) superfamily and evaluated their prognostic values in serous ovarian cancer patients.

**Methods:**

We first analyzed the differential expression of SIRT members among fallopian tube epithelium (FTE), primary serous ovarian cancers/tubal cancers (PSOCs/PSTCs), and omental metastases using GSE10971 and GSE30587 datasets. The prognostic values of SIRT members were evaluated using TCGA and GSE9891 dataset.

**Results:**

SIRT3 and SIRT5 expression were significantly decreased and increased in PSOCs/PSTCs compared with that in normal counterparts, respectively. SIRT6 and SIRT7 were overexpressed in ometal metastases compared with corresponding primary counterparts. With respect to recurrence free survival, however, SIRT7 overexpression was correlated with better prognosis. A similar trend was observed by multivariable analysis. Regarding overall survival (OS), increased expression of SIRT3, SIRT5, and SIRT7 were associated with better survival by univariable analysis. Subsequent multivariable analysis showed that SIRT3 remained an independent favorable prognostic factor for OS. The SIRT3-related nomogram illustrated age at initial diagnosis as sharing the largest contribution to OS, followed by SIRT3 expression and FIGO stage. The C-index for OS prediction was 0.65 (95%CI, 0.61–0.69) in training cohort (TCGA dataset) and 0.65 (95%CI, 0.59–0.71) in validation cohort (GSE9891 dataset), respectively. The calibration plots showed optimal agreement between the prediction by SIRT3-related nomogram and actual observation for 1-, 3-, and 5-year OS probability.

**Conclusion:**

In conclusion, SIRT3 was an independent favorable prognostic factor for OS in serous ovarian cancer, and added prognostic value to the traditional clinicopathological factors used to evaluate patients’ prognosis.

## Introduction

Ovarian cancer, characterized by late-stage presentation and metastatic bulky disease burden, is the leading cause of gynecologic cancer death [1]. After initial cytoreductive surgery combined with platinum based chemotherapy, most of the patients who achieving complete remission still face a high risk of recurrence and death, and their 5 year survival rate is ranged from 20 to 30%. Thus, it is important to identify new biomarkers to predict ovarian cancer prognosis, which will facilitate timely inclusion into clinical trials or personalized treatment strategies.

Sirtuins (SIRTs), initially identified as adenosine diphosphate (ADP) ribosyltransferases in bacterium *Salmonella typhimurium*, are highly conserved histone deacetylases (HDAC) dependent on nicotine adenine dinucleotide (NAD) and/or mono ADP-ribosyltransferases [2, 3]. Currently, a superfamily of seven SIRTs (SIRT1–7) has been identified in mammalian, and each has its own unique characteristics and functions. SIRT1, SIRT6 and SIRT7 are predominantly localized in the nucleus; SIRT3, SIRT4 and SIRT5 are mitochondrial proteins and primarily reside within the mitochondria; and SIRT2 is localized mainly in the cytoplasm [3]. In certain conditions, SIRT1 and SIRT2 are able to shuttle between the nucleus and cytoplasm.

SIRTs received increasing attention due to their divergent role in cancer biology. The role of SIRTs is of extreme complexity, with both tumor promoter and tumor suppressor functions dependent on cell contexts. In ovarian cancer, SIRTs has been implicated in the regulation of various cellular processes, including but not limited to cell growth, apoptosis, invasion, and metastasis [4–10]. However, the role of SIRTs in predicting progression and prognosis of ovarian cancer patients are still largely elusive.

In this study, we analyzed the expression pattern of the SIRT superfamily (SIRT1-SIRT7) and evaluated their prognostic values in serous ovarian cancer patients by using the high-throughput expression data deposited in Gene Expression Omnibus(GEO) database and The Cancer Genome Altas (TCGA) database.

## Materials and methods

### Databsets used in present study

The publicly available gene expression data used in our study is described in TCGA and GEO database(GSE10971, GSE30587 and GSE9891 datasets). GSE10971 dataset was used to determine the differential expression of SIRT members between fallopian tube epithelium (FTE) (*n* = 24) and primary serous ovarian cancer/tubal cancers (PSOCs/PSTCs) (*n* = 13). GSE30587 dataset was used to determine the differential expression of SIRT members between paired PSOC tissues(*n* = 9) and their omental metastasis counterparts(n = 9). TCGA dataset was used to explore the association of SIRT members’ expression with clinicopathological factors and to develop SIRT-related nomograms to predict recurrence free survival (RFS) and overall survival (OS). GSE9891 dataset was used to validate the nomograms developed from TCGA dataset. For both TCGA and GSE9891 dataset, only serous ovarian cancer patients with adequate information (including age at initial diagnosis, histological grade, FIGO stage, debulking status, days to recurrence, recurrence status, days to death, and vital status) were included in our analysis. Collectively, there were 462 patients in TCGA dataset and 226 patients in GSE9891 dataset included in our final analysis. TCGA dataset and GSE9891 dataset were obtained from the “curatedOvarianData” Bioconductor package (version 2.12 for R 3.0.3).

### Statistical analysis

Continuous data between two groups were compared using two independent samples t test. Categorical data were compared using chi-square or Fisher’s exact test where appropriate. Both univariable and multivariable analyses were performed in survival analysis. The statistical analyses mentioned above were analyzed using IBM SPSS Statistics (version 22.0). *P* values < 0.05 were considered significant (*P* < 0.05).

We developed the SIRT-related nomogram using rms package. Harrell’s concordance index (c-index) was calculated through a bootstrap method with 1000 resamples and used to evaluate the predictive efficiency of nomogram. Calibration curves were used to compare the prediction by nomogram and actual observation graphically. The statistical analyses mentioned above were analyzed using R (version 3.5.2). *P* values < 0.05 were considered significant (*P* < 0.05).

## Results

### The expression pattern of SIRT family members in serous ovarian cancer

**Fig. 1:**
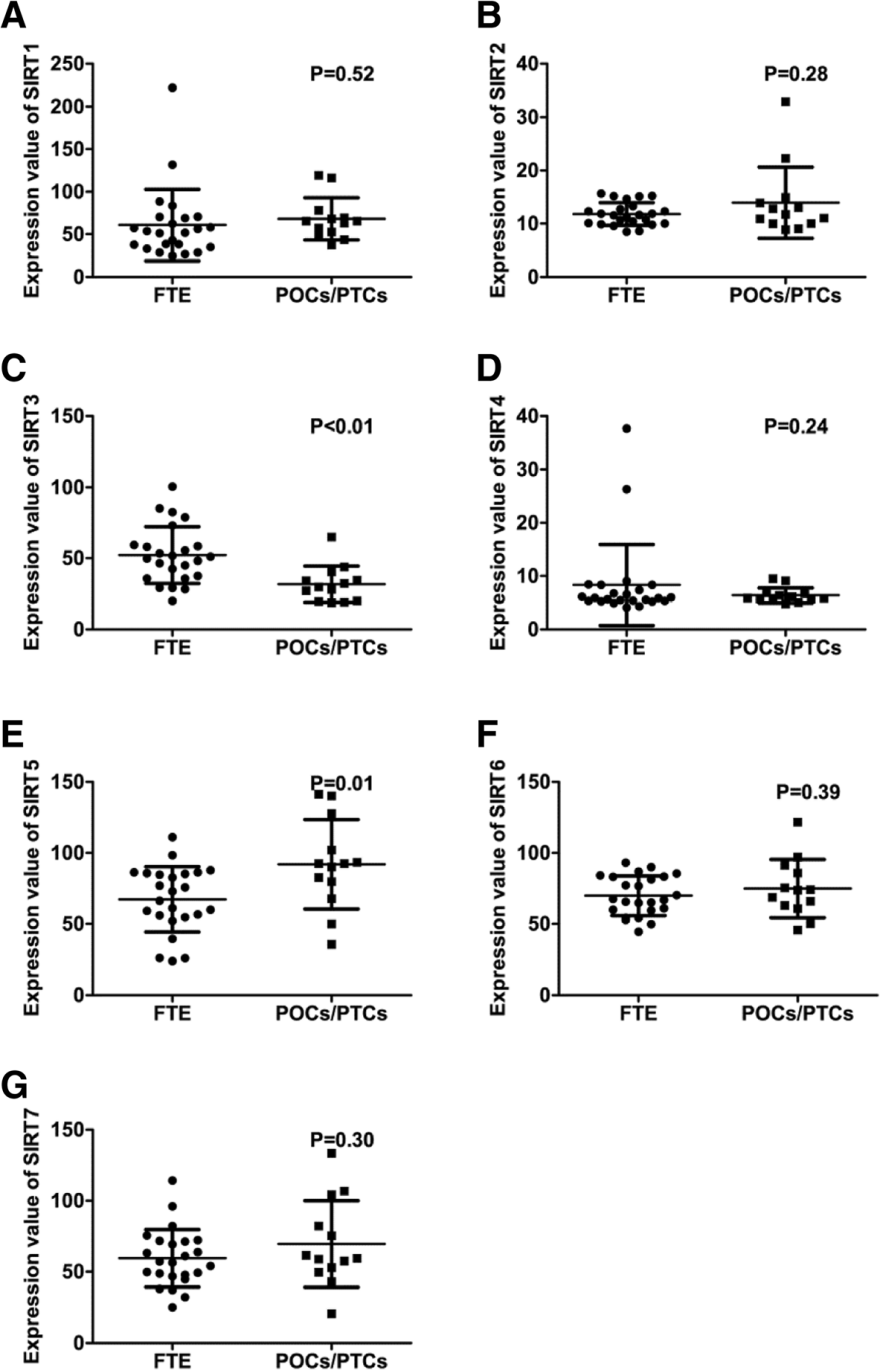
Differential Expression of SIRT members between FTE and PSOCs/PSTCs. (**a**-**g**) There was no significant difference in the expression of SIRT1 (**a**), SIRT2 (**b**), SIRT4 (**d**), SIRT6 (**f**), and SIRT7 (**g**) between FTE and PSOCs/PSTCs. SIRT3 (**c**) and SIRT5 (**e**) expression were significantly decreased and increased in PSOCs/PSTCs compared with that in normal counterparts, respectively

**Fig. 2:**
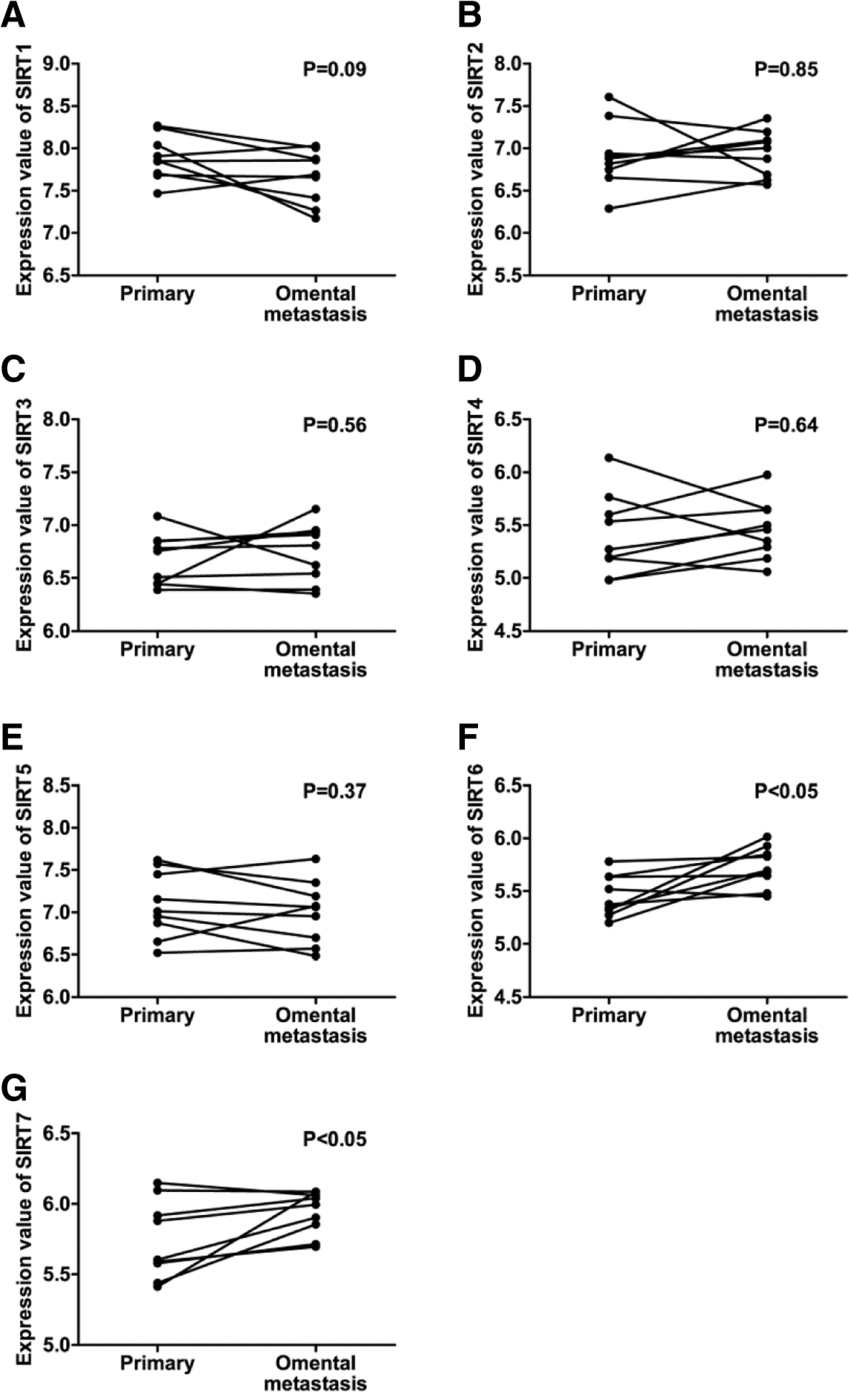
Differential Expression of SIRT members between PSOCs and paired omental metastases. (**a**-**g**) There was no significant difference in the expression of SIRT1 (**a**), SIRT(**b**), SIRT3 (**c**), SIRT4 (**d**), and SIRT5 (**e**) between PSOCs and paired omental metastases. SIRT6 (**f**) and SIRT7 (**g**) were significantly overexpressed in ometal metastases compared with corresponding primary counterparts

We first determined the differential expression of SIRT family members between FTE and PSOCs/ PSTCs. We found that SIRT3 expression was significantly decreased in PSOCs/PSTCs compared with that in FTE (Fig. 1C), whereas SIRT5 was obviously increased in PSOCs/PSTCs compared with that in FTE (Fig. 1E). The expression of the rest members of SIRT family was comparable between FTE and PSOCs/PSTCs (Fig. 1A, B, D, F, G). Then, GSE30587 dataset was used to determine the differential expression of SIRT members between paired PSOC tissues and their omental metastasis counterparts. It was found that SIRT6 and SIRT7 were overexpressed in ometal metastases compared with corresponding primary counterparts (Fig. 2F, G). The expression of the rest members of SIRT family was comparable between PSOCs and correponding omental metastases (Fig. 2A-E).

### The prognostic value of SIRT members in serous ovarian cancer patients

Next, we evaluated the prognostic value of each SIRT member in predicting RFS and OS in serous ovarian cancer patients using the TCGA dataset. The results of the univariable and multivariable analysis are listed in Table 1. With respect to RFS, univariable analysis showed that increased FIGO stage decreased SIRT7 expression were correlated with worse prognosis. A similar trend was observed by multivariable analysis (Table 1). Regarding OS, younger age at initial diagnosis was associated with better prognosis. Increased FIGO stage, high grade, and suboptimal debulking had a negative impact on survival. With respect to SIRT members, increased expression of SIRT3, SIRT5, and SIRT7 were all correlated with better survival. All factors reaching statistical significance in the univariable analysis were entered into the multivariable Cox regression analysis. Age at initial diagnosis, FIGO stage, and SIRT3 expression remained independent prognostic factors for OS (Table 1).

**Table 1:** The associations of each SIRT member’s expression with RFS and OS

Variables	Number of patients	RFS				OS			
		Univariate analysis		Multivariate analysis		Univariate analysis		Multivariate analysis	
		HR (95%CI)	*P* value	HR (95%CI)	*P* value	HR (95%CI)	*P* value	HR (95%CI)	*P* value
Age	462	1.003 (0.991–1.014)	0.661	/	/	1.024 (1.013–1.036)	< 0.001	1.023 (1.011–1.034)	< 0.001
Stage			0.015		0.016		0.001		0.005
Early	33	1		1		1		1	
Late	429	2.003 (1.144–3.506)		1.986 (1.134–3.477)		3.478 (1.639–7.381)		2.995 (1.392–6.446)	
Grade			0.254		/		0.048		0.196
Low	57	1		/		1		1	
High	405	1.227 (0.863–1.745)		/		1.438 (1.003–2.063)		1.280 (0.881–1859)	
Debulking			0.886		/		0.030		0.540
Optimal	344	1		/		1		1	
Suboptimal	118	0.979 (0.734–1.306)		/		1.353 (1.030–1.776)		0.915 (0.689–1.216)	
SIRT1	462	0.824 (0.662–1.026)	0.084	/	/	0.907 (0.727–1.131)	0.386	/	/
SIRT2	462	1.061 (0.814–1.384)	0.660	/	/	1.187 (0.925–1.522)	0.178	/	/
SIRT3	462	1.043 (0.690–1.576)	0.841	/	/	0.661 (0.438–0.996)	0.048	0.588 (0.389–0.889)	0.012
SIRT4	462	0.629 (0.385–1.028)	0.064	/	/	0.627 (0.374–1.052)	0.077	/	/
SIRT5	462	1.013 (0.856–1.199)	0.879	/	/	0.815 (0.688–0.967)	0.019	0.882 (0.742–1.049)	0.157
SIRT6	462	1.102 (0.697–1.744)	0.677	/	/	0.794 (0.505–1.249)	0.318	/	/
SIRT7	462	0.786 (0.620–0.997)	0.047	0.791 (0.624–1.003)	0.053	0.760 (0.595–0.969)	0.027	0.786 (0.613–1.008)	0.058

### Development and validation of SIRT3-related nomogram predictive of OS

**Fig. 3:**
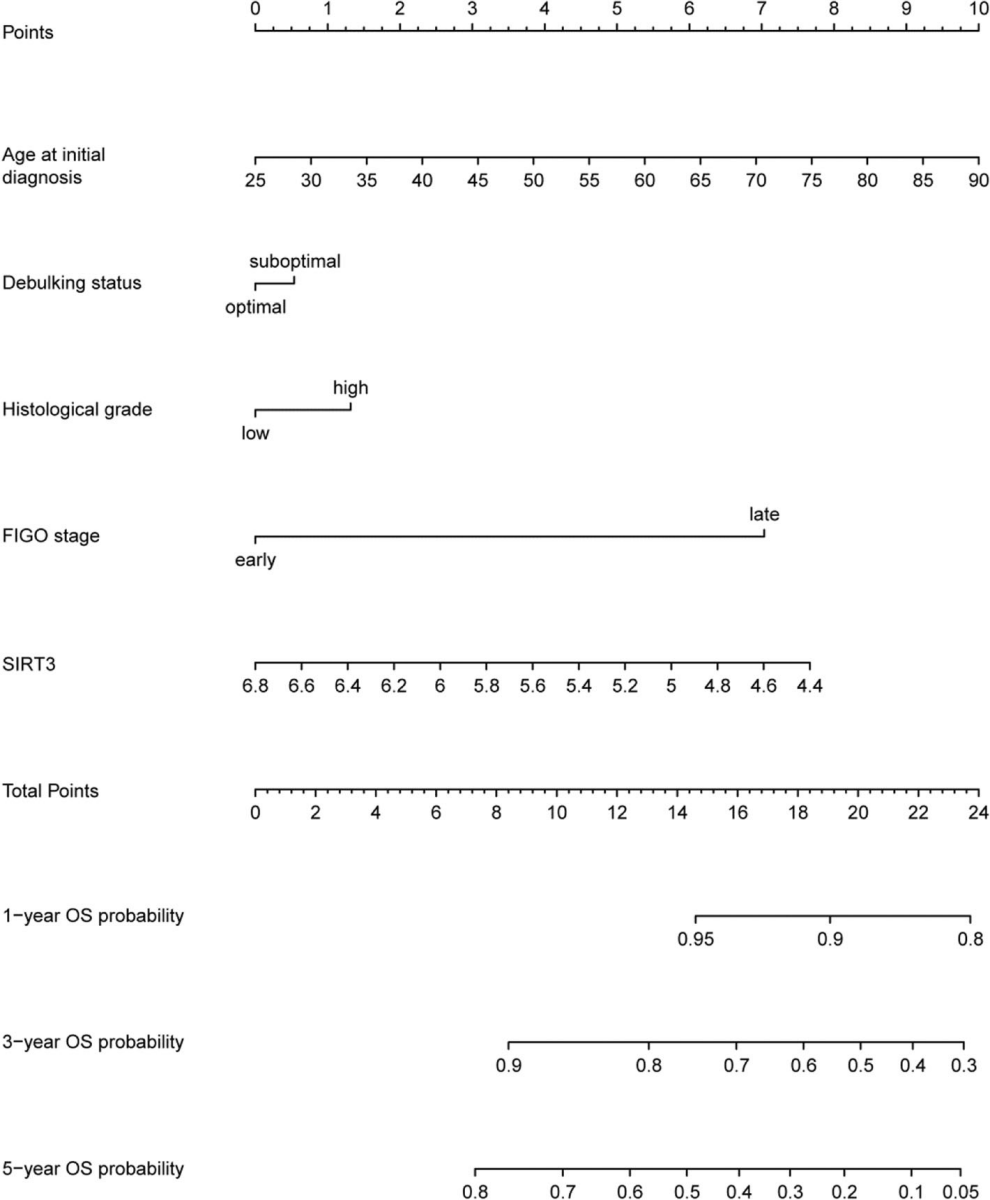
The SIRT3-related nomogram developed from TCGA dataset. The nomogram illustrated age at initial diagnosis as sharing the largest contribution to OS, followed by SIRT3 expression and FIGO stage. Histological grade and debulking status had a moderate impact on the OS

**Fig. 4:**
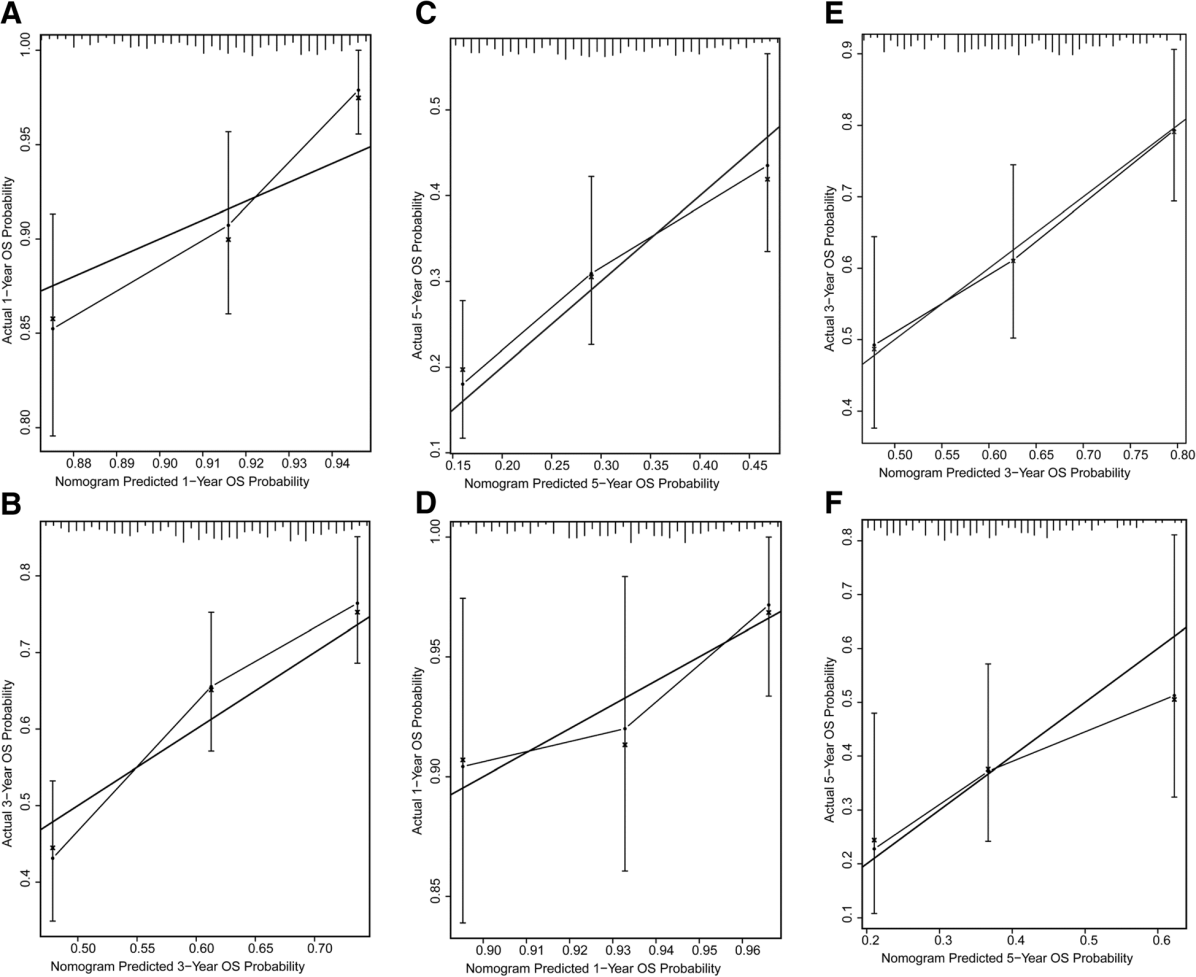
Calibration plots of the SIRT3-related nomogram to predict OS. (**a**-**c**) Calibration curves of the SIRT3-related nomogram to predict OS at 1 year (**a**), 3 years (**b**), and 5 years (**c**) in TCGA dataset. (**d**-**f**) Calibration curves of the SIRT3-related nomogram to predict OS at 1 year (**d**), 3 years (**e**), and 5 years (**f**) in GSE9891 dataset

To precisely predict the OS of serous ovarian cancer patients, we tried to develop a prognostic nomogram. Besides independent prognostic factors indicated by multivariable analysis, another two established prognostic factors, including histological grade and debulking status, were also incorporated to develop the prognostic nomogram (Fig. 3). The nomogram illustrated age at initial diagnosis as sharing the largest contribution to OS, followed by SIRT3 expression and FIGO stage. Histological grade and debulking status had a moderate impact on the OS. The C-index for OS prediction was 0.65 (95%CI, 0.61–0.69). The calibration plots showed optimal agreement between the prediction by nomogram and actual observation for 1-, 3-, and 5-year OS probability in TCGA dataset (Fig. 4A-C).

GSE9891 dataset was used as a validation cohort. The C-index for OS prediction was 0.65 (95%CI, 0.59–0.71) in GSE9891 dataset. The calibration plots also showed optimal agreement between the prediction by nomogram and actual observation for 1-, 3-, and 5-year OS probability (Fig. 4D-F).

## Discussion

The expression and prognostic value of some SIRT members have been explored in ovarian cancer. However, the small sample sizes in previous studies have yielded inconsistent results, raising doubts in generalizing their findings. In this study, we explored the expression and clinical significance of all the SIRT members in serous ovarian cancer with a larger sample size by using high throughput mRNA profile datasets from both TCGA database and GEO database.

Among the SIRT family, SIRT1 is the most extensively studied member. However, its role in ovarian cancer are still uncertain. Ray et al. indicated that increased expression and activity of SIRT1 impaired the invasion of ovarian cancer cells by targeting the EMT inducer ZEB1 [7]. Downregulation of SIRT1 also led to decreased migration and angiogenesis of ovarian cancer cells by repressing HMGB1 [5], and was associated with decreased OS in serous ovarian cancer. However, in contrast, other studies showed that SIRT1 overexpression or activation resulted in acquired chemoresistance and increased invasiveness of ovarian cancer cells [8, 11], and was associated with a poorer prognosis in serous ovarian cancer patients [10]. Our data indicated that SIRT1 expression appeared to be downregulated in omental metastasis compared with primary couterparts, and was associated with extended RFS in TCGA dataset though without reaching statistical significance.

SIRT3 is a well known and the best characterized mitochondrial sirtuin (mtSIRT) and capable of regulating multiple major aspect of mitochondrial biology [12]. The role of SIRT3 in cancer biology is still in debate [13, 14]. The expression of SIRT3 was aberrantly decreased in head and neck squamous cell carcinoma (HNSCC), gastric cancer, and mantle cell lymphoma [15–17]. However, in certain cancers, SIRT3 were significantly upregulated [18–20]. Whereas in breast cancer, contradictory results have been observed [21–23]. In ovarian cancer, SIRT3 expression was significantly downregulated in cancer tissues as demonstrated by both our study and a previous study [24]. Moreover, SIRT3 was also downregulated in the metastatic tissues and highly metastatic cell line of ovarian cancer [9]. Upregulation of SIRT3 impaired the viability, migration, and invasion of ovarian cancer cells [4, 9, 24]. Similar to the dual roles of SIRT3 in cancer biology, the prognostic value of SIRT3 in cancer is also dichotomous. Some studies indicated that decreased expression of SIRT3 was associated with poor prognosis in pancreatic, gastric, and hepatocellular cancer [25, 26]. In certain cancer, in contrast, increased expression of SIRT3 was correlated with a worse clinical outcome [27]. However, the prognostic value of SIRT3 in ovarian cancer has not yet been evaluated. In present study, we found that SIRT3 is an independent predictor of better OS in serous ovarian cancer. Moreover, our SIRT3-related nomogram indicated that SIRT3 shared a larger contribution to OS, compared with FIGO stage, histological grade, and debulking status.

In contrast to SIRT3, the roles of the other two mtSIRTs, SIRT4 and SIRT5, are not yet explored in ovarian cancer, though their dual puzzling functions as tumor suppressors and tumor promoters have been reported in other cancers [28–30]. In ovarian cancer, It seemed that increased expression of SIRT4 was associated with a better prognosis by our univariable analysis, though without reaching statistical significance. Whereas SIRT5 appeared to a risk factor for worse prognosis. Their functions in ovarian cancer remains to be explored in future studies.

The roles of SIRT 6 and SIRT7 in cancer were also cellular context dependent. Some studies indicated that SIRT6 could inhibit ovarian cancer cell growth and was a favorable prognostic factor [31, 32]. However, Bae et al. indicated SIRT6 promoted invasion and migration of ovarian cancer cells, without affecting cell proliferation [6]. Moreover, SIRT6 was associated with higher FIGO stage, higher histological grade, platinum-resistance, and shorter OS [6]. Consistently, our work revealed that SIRT6 was elevated in omental metastases compared with paired PSOCs. However, survival analysis failed to demonstrate a prognostic value of SIRT6 in serous ovarian cancer. With respect to SIRT7, it was reported that SIRT7 was unregulated in ovarian cancer cells compared with normal couterparts, and could promote cell growth and colony formation, and reduce cell apoptosis [33]. Our data suggested that SIRT7 was upregulated in omental metastases compared with paired PSOCs. However, our univariable analysis revealed that SIRT7 overexpression was associated with both better RFS and OS. It seemed that SIRT6 and SIRT7 displayed both oncogenic and tumor-suppressive properties in ovarian cancer.

In conclusion, SIRT3 was an independent favorable prognostic factor for OS in serous ovarian cancer, and added prognostic value to the traditional clinicopathological factors used to evaluate patients’ prognosis.
